# Inducing Cough Reflex by Capsaicin Spray Stimulation in Patients with Acquired Brain Injury: A Preliminary Test and Proof of Concept

**DOI:** 10.3390/clinpract13060140

**Published:** 2023-12-15

**Authors:** Luisa Spezzano, Maria Daniela Cortese, Maria Quintieri, Loris Pignolo, Paolo Tonin, Francesca Lucia Lucca, Francesco Tomaiuolo, Rocco Salvatore Calabrò, Giovanni Morone, Antonio Cerasa

**Affiliations:** 1 S’Anna Institute, 88900 Crotone, Italy; l.spezzano@isakr.it (L.S.); d.cortese@isakr.it (M.D.C.); m.quintieri@isakr.it (M.Q.); l.pignolo@isakr.it (L.P.); patonin18@gmail.com (P.T.); l.lucca@istitutosantanna.it (F.L.L.); 2 Department of Clinical and Experimental Medicine, University of Messina, 98122 Messina, Italy; francesco.tomaiuolo@gmail.com; 3 IRCCS Centro Neurolesi “Bonino-Pulejo”, 98123 Messina, Italy; salbro77@tiscali.it; 4 Department of Life, Health and Environmental Sciences, University of L’Aquila, 67100 L’Aquila, Italy; giovanni.morone@univaq.it; 5 San Raffaele Sulmona Institute, 67039 Sulmona, Italy; 6 Institute for Biomedical Research and Innovation (IRIB), National Research Council of Italy (CNR), 98164 Messina, Italy; 7 Pharmacotechnology Documentation and Transfer Unit, Preclinical and Translational Pharmacology, Department of Pharmacy, Health and Nutritional Sciences, University of Calabria, 87036 Rende, Italy

**Keywords:** cough reflex, acquired brain injury, capsaicin, dysphagia

## Abstract

Individuals with acquired brain injuries (ABIs) may experience various complications related to poor coughing or impaired cough reflex (including risk of aspiration pneumonia or respiratory infections). For this reason, cough assessment is an important component in the clinical evaluation since patients with ABI are not able to cough voluntarily due to severe motor deficits. When voluntarily coughing is not possible, it is essential for clinical practices to find a quick and minimally invasive way to induce a cough reflex. In the present study, we evaluated the cough reflex in ABI patients using a new method based on a capsaicin spray stimulation test. In total, 150 healthy controls demographically matched with 50 ABI patients were included in this study. Clinical observations demonstrated robust cough response in both healthy controls and ABI patients, as well as the safety and tolerability of capsaicin spray stimulation. ABI patients with dysphagia were characterized by slower and delayed cough responses. Further studies are needed to validate this feasible, less-invasive, and simple-to-comprehend technique in inducing cough reflex. According to this preliminary evidence, we believe that this test might be translated into a simple and effective treatment to improve reflexive cough modulation in ABI patients.

## 1. Introduction

Cough reflex deficits can occur in those individuals who have experienced a neurological event that may alter the afferent, central, or efferent components of this reflex temporarily (such as after general anesthesia) or permanently (such as after a stroke, cervical cord damage, Parkinson’s disease, or amyotrophic lateral sclerosis) [1,2,3]. This can occur due to damage to or dysfunction of any structure that is involved in the cough mechanism, including the medulla oblongata. In these cases, the patient may have difficulty initiating a cough and may be at risk for aspiration and pulmonary infections [4]. Treatment options may include the use of assisted coughing techniques, such as the use of an incentive spirometer or vibration therapy, and/or the use of medications to help stimulate the cough reflex [5,6].

The cough reflex test can be used as a screening tool in clinical practice to evaluate the integrity of the cough response to airway invasion and the potential for silent aspiration [7]. It includes a nebulizer to inhale a cough-evoking aerosol at a particular concentration in order to elicit coughing. In addition to self-reported ratings of the perceived severity of airway irritation, clinicians can note the presence, absence, and quantity of coughs that are evoked. It offers information that would not otherwise be available on the consistency of upper airway sensation. One factor raising patients’ risk of silent aspiration is impaired upper airway feeling. A patient with impaired upper airway feeling may not aspirate if their swallowing biomechanics are not altered. Therefore, it is necessary to interpret the cough reflex test results considering the full clinical swallowing evaluation. On instrumental examination, citric acid is used to test for silent aspiration [7]. This test exposes the patient to a small amount of citric acid measuring the response, including the time to onset of coughing and the number of coughs produced. Other commonly reported cough-evoking techniques are the nebulized 2.5% hypertonic saline, epinephrine, or mannitol [8].

The capsaicin inhalation test is another method used to evaluate the cough reflex in neurological patients [7,8]. This is currently the most widely utilized non-acid tussive substance in cough inhalation tests. Several guidelines, including those from the Chinese Thoracic Society (CTS), European Respiratory Society (ERS), and American College of Chest Physician (ACCP), recommend the use of capsaicin inhalation challenges [9,10]. Capsaicin is a flavorful compound with the chemical formula C_18_H_27_NO_3_ found in chili peppers, which can cause reflex coughing in humans. Since the 1980s, the test has been performed by having the patient inhale a small amount of capsaicin through a *nebulizer* [11,12]. The patient’s coughing response is then measured and recorded, including the time to onset of coughing and the number of coughs produced. The test can be used to evaluate the patient’s ability to produce an effective cough and to detect any underlying problems with the cough reflex, with a documented excellent safety record in healthy volunteers as well as in patients with asthma, chronic obstructive pulmonary disease, pathologic cough, and other respiratory conditions [13,14]. This test has been applied either for the diagnosis of cough reflex deficits or for therapeutic purposes in patients with neurogenic dysphagia [15], progressive supranuclear palsy [16], Parkinson’s disease [17], and in patients with hemorrhagic stroke [18].

The wide range of techniques described in the literature presents one of the biggest obstacles to integrating the cough reflex test into clinical practice. In particular, in patients with acquired brain injury (ABI), such as stroke and traumatic or anoxic injury, there is no defined standard procedure (i.e., nebulizer flow rates, duration and concentration of substances exposure) to evaluate cough reflex. Therefore, in a clinical neurological setting, a feasible, less-invasive, less time-consuming/labor-consuming, and simple-to-comprehend technique is advantageous.

For this reason, in this preliminary study, we sought to evaluate the effectiveness of a different kind of simple and timesaving cough reflex induction method based on a commercial capsaicin spray stimulation applied directly on the tongue of patients with ABI. We believe that this test could be easy to apply and time-sparing and could have good sensitivity and reproducibility among clinicians.

## 2. Methods

### 2.1. Participants

**Figure 1:**
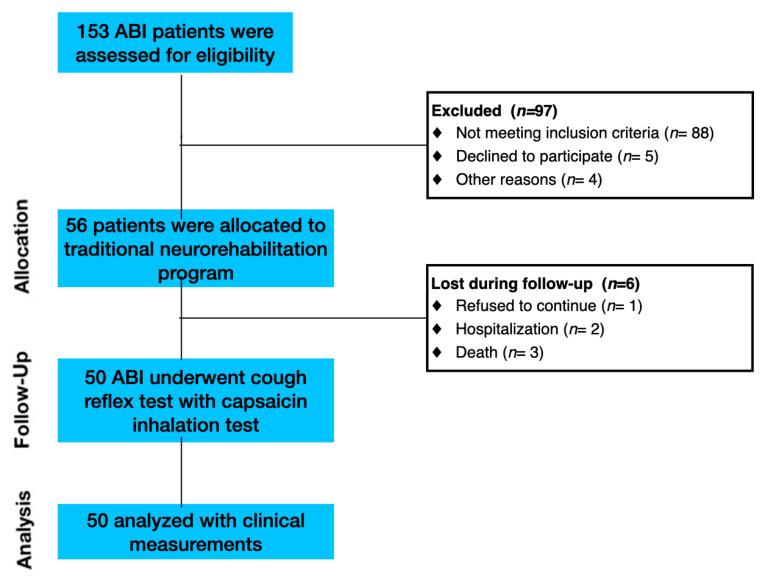
Flow diagram of participant recruitment and participation in the study.

The study involved patients affected by ABI. Inpatients consecutively admitted to the Intensive Rehabilitation Unit (IRU) of the Institute S. Anna (Crotone, Italy) between January 2020 and December 2022 were screened for possible inclusion. From an initial cohort of 153 ABI patients, we enrolled only ABI patients who fulfilled the following inclusion criteria: (1) age ≥ 18 years; (2) first admission to the neurorehabilitation unit; (3) Level of Cognitive Functioning Scale (LCF ≥ 4); and (4) ability to perform three trials of capsaicin stimulation. The exclusion criteria included: (1) history of asthma or other respiratory conditions; (2) patients with active respiratory infection; and (3) hypersensitivity to low levels of capsaicin. Based on the inclusion and exclusion criteria, 103 ABI patients were excluded (Figure 1).

Healthy controls (HCs) were recruited from universities, community recreational centers and hospital personnel through local advertisements. Inclusion criteria were as follows: 1) no evidence of neuro logical and psychiatric symptoms according to DSM-V criteria; (2) no use of antidepressant, anxiolytic, or antipsychotic drugs; (3) absence of chronic medical conditions (i.e., history of asthma or other respiratory conditions); and (4) no smokers. Based on the inclusion and exclusion criteria, 150 HCs were enrolled.

All patients and/or their caregivers gave written informed consent, and the study was approved by the Ethical Committee of the Central Area Regione Calabria of Catanzaro (CZ-Prot 24-2019), according to the Helsinki Declaration.

### 2.2. Design and Procedure

All the included HCs and patients underwent preliminary baseline reflex and voluntary cough clinical evaluations. The Gugging Swallowing Screen (GUSS) scale was used at admission to determine the dysphagia severity [19] in ABI patients. The GUSS scale consists of two parts: the indirect swallowing test and the direct swallowing test. The higher the final score, the better the performance. The total score ranges from 0 to 20 and is subdivided as follows: 0 to 9 means that the pretest or ingestion of semisolid food failed and the dysphagia is classified as severe with a high risk of aspiration; 10 to 14 means that the patient swallows semisolid food without difficulty but has difficulty with liquids and the dysphagia is classified as moderate with a low risk of aspiration; 15 to 19 means there was no difficulty in swallowing semisolid or liquid foods, while there was difficulty with solid foods, so dysphagia is rated as mild with minimal risk of aspiration; and 20 corresponds to the absence of difficulty, so mild/no dysphagia with minimal risk of aspiration was noted. This scale allows us to stratify the severity of dysphagia and also detect patients without dysphagia.

**Figure 2:**
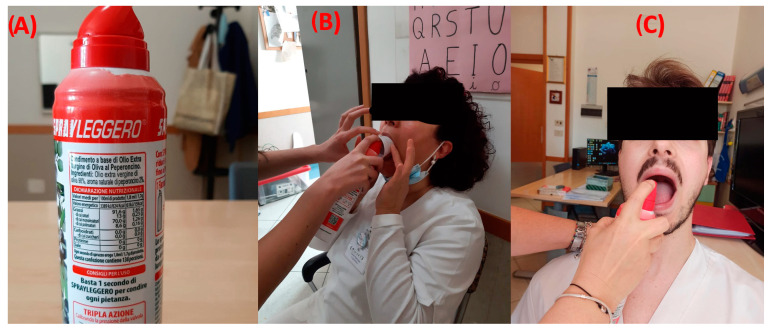
Chili pepper administration. (**A**) Spray; (**B**) administration in the case of patients with trouble opening their mouths (they can receive treatment by a tube while their mouth is held open); (**C**) traditional administration directly on the patient’s tongue.

After admission to the IRU (before starting standard rehabilitation protocols), the enrolled patients were tested with our cough reflex test (called the Chili Pepper Test), as the first component of a standard bedside swallow examination. All patients were examined only after the decannulation. A spray oil that contains capsaicin was used for the test (Compagnia Alimentare Italiana s.p.a.; Broccostella (FR); 03030; Italy; https://sprayleggero.it/en/ (accessed on 1 January 2021)). The stimulation protocol requires three maximum doses of capsaicin. During the stimulation, the subject’s nose was pinched closed. Open-mouthed, on the posterior third of the tongue, a spray of oil containing 1.7 g of 2% capsaicin equivalent dose was applied (Figure 2). To prevent interfering with the perception of acidity and saltiness in the anterior two-thirds of the tongue, capsaicin was only sprayed in the central posterior third. We waited from 0 to 60 s after stimulation to note the presence or absence and the duration of the cough response’s latency. After the first administration (0.034 g), a second stimulation with a double concentration (0.068 g) was made only in the absence of a first response. If no reactivity was noticed, a third and final dose with a tripled concentration (1.02 g) was then given. The expected result of a normal cough reflex test was an immediate series of coughs, which are primarily expiratory “airway clearing” in character. If the subject had a normal cough reflex after the first administration, additional stimulations were not performed. Each single spray dose corresponds to the basic capsaicin concentration (1.8 mL/1.7 g)

The cough reflex test was performed by one clinically certified speech language pathologist, with more than 10 years of experience rating swallows and cough reflexes in ABI patients (L.S.).

### 2.3. Outcome Measures

An additional ad hoc questionnaire was included in the protocol to assess the intensity of cough reflex after the three capsaicin administrations. The questionnaire was structured as a Likert scale and included 4 items: 0–3 multiple answers, scored from the lowest to highest intensity level of cough reflex (*0 “absent”—no cough reflex; 1 “feeble”—cough reflex present but ineffective; 2 “wheezing”—cough reflex present and bothersome; 3 “persistent/irritating”—persistent cough reflex with duration > 5 s*). One expert clinician with more than 20 years of experience in brain injury performed outcome measurements.

### 2.4. Statistical Analysis

Statistical analysis was performed using the Statistical Package for Social Science software (SPSS, v20.0, Chicago, IL, USA) for Macintosh. Assumptions of normality were tested for all the continuous variables. Normality was tested using the Kolmogorov–Smirnov test. X^2^, unpaired *t*-test, and One-Way ANOVA were used to evaluate the differences in demographic and clinical factors between groups, and to evaluate significant changes detected during the cough reflex test. For all the tests, a *p*-value < 0.05 was statistically significant.

## 3. Results

### 3.1. Clinical Data

There was no difference between patients and HCs concerning age (50.6 ± 12.7 years HCs; 55.5 ± 18.1; *t*-test = 2.2; *p*-level = 0.09) and gender distribution (male: 54% HCs; male: 65% ABI; X^2^ = 1.5; *p*-level = 0.23). At admission, 24 ABI patients had dysphagia (58% characterized by a severe condition according to GUSS). For this reason, we decided to group patients according to this critical symptom.

Table 1 shows the demographic characteristics of ABI patients with or without severe dysphagia. At admission, ABI patients with dysphagia were characterized by a different clinical condition starting with an almost significant distribution of etiology (X^2^ = 5.1; *p* = 0.06). In particular, these patients had a longer stay in ICU (37.1 ± 17.7 days), right-side prevalence of brain lesions (42%), higher prevalence of PEG (46%), tracheostomy (96%), and poorer clinical status (Barthel Index = 2.7 ± 9.4; GUSS = 8.9 ± 4.2) with respect to ABI without dysphagia (24.2 ± 15.3 days, 12%; 0%; 4%; 13.2 ± 20.1; *t* = 2.4 *p* < 0.02; X^2^ = 10.1; *p* < 0.006; X^2^ = 6.4; *p* < 0.04; X^2^ = 98.1; *p* < 0.001; *t* = 2.89; *p* < 0.006; *t* = 8.98; *p* < 0.001; respectively).

### 3.2. Cough Reflex Test

No adverse events or parasympathetic responses were detected in HCs and patients. Descriptive statistics for cough reflex response and parameters are included in Table 2.

As concerns the presence of a cough reflex, there was no significant difference among the three groups. ABI patients without dysphagia showed the most sensitive response to tongue stimulation (77% with relevant cough reflex at 1° administration), with respect to the HCs group (68%) and ABI patients with dysphagia (50%). Both HCs (post hoc *t*-test; *p*-level = 0.008) and ABI patients without dysphagia (post hoc *t*-test; *p*-level = 0.02) showed the fastest response (12.2 and 12.1 s, respectively) with respect to ABI patients with dysphagia (23.8 s; F-test = 3.4; *p*-level = 0.049). Finally, the cough intensity was feeble and similar among the three groups.

## 4. Discussion

This study aimed to evaluate the effectiveness of a new kind of capsaicin stimulation test to evaluate the cough reflex in patients with ABI. According to the current pilot investigation, our capsaicin stimulation test consistently causes coughing in healthy volunteers as well as in ABI cohorts with or without dysphagia (all post hoc *t*-test > 0.05). However, a slower and delayed cough response was detected in ABI patients with dysphagia with respect to HCs and ABI without dysphagia.

It is crucial to emphasize that distinct cough reflex reactions are caused by different nebulizers, speed, and tussigenic substances [13]. The approaches used for the cough reflex test have varied among different research, which may be the potential cause of the significant differences in the methods and may prevent fair comparison [20]. Basically, the two most frequently employed tussigenic substances reported in cough-evoking aerosols literature are capsaicin and citric acid. Citric acid, in contrast to capsaicin, has been shown to activate both chemoreceptors and mechanoreceptors [10]. Laryngeal coughing, instead, is reported to be induced by citric acid [13]. The choice of aerosol should be carefully considered, as it has implications for the underlying neurophysiology of the induced cough. Citric acid preferentially stimulates neural pathways and rapidly adapts laryngeal receptors that play a role in coughing to aspiration [21]. Capsaicin preferentially stimulates slowly adapting sensory receptors that mediate coughing to prolonged airway irritation. With respect to previously reported cough-evoking aerosol methods we decided to use a commercial spray solution in order to directly stimulate the oropharynx tract. Indeed, it is generally known that vagal afferents that innervate the larynx, trachea, and airways mediate the cough reflex. The glossopharyngeal nerve in the nasopharynx supplies sensory innervation to the pharynx, although the oropharynx and laryngopharynx get dual innervation from the glossopharyngeal and vagus nerves. Therefore, it is conceivable that oropharynx-related treatments may affect coughing by affecting the local vagal afferent fibers. Furthermore, there is evidence that the cough reflex can also be mechanically elicited from the pharynx, even though mechanical stimulation of the oropharynx can cause adverse effects [22]. The larynx and esophagus’ vagal afferent fibers are additional potential sites of action. Therefore, we believe that the effect of capsaicin spray on the oropharynx in our study suggests that the activation of pharyngeal sensory fibers plays a different role in contributing to cough reflex with respect to previously reported cough-evoking aerosol methods.

Two important findings of our study need to be highlighted. First, the Cough Reflex Trigger time is longer in patients with dysphagia, and the response to the first stimulation is reduced in patients with dysphagia compared to in ABI patients without dysphagia and HCs. This is because, as evidenced by the Barthel Index at admission, patients with dysphagia are more severe than those without (see Table 1). This is an important observation that gives clinicians the opportunity to understand that the cough reflex in patients with dysphagia can be evoked over the three administrations and is of valid intensity but has a delayed trigger. This should advise therapists and phoniatrics of the risk of pre-swallowing falls with the risk of inhalation and the consequent probability of aspiration pneumonia. Next, during our experimental procedures, no adverse events were detected. This is coherent with the current literature that has described only a skin facial irritation that is self-limited without the use of drugs [12,23,24]. Moreover, according to a previous study, no alteration of the heart rate, respiratory rate, or oxygen saturation was observed during capsaicin administration [18].

### Limitations

We acknowledge that the main issue with this study is the absence of an instrumental assessment, as well as the lack of intra/inter-rater evaluation of cough response. Indeed, we could have measured the intensity and duration of cough reflex using spirometry. Although our primary goal was to realize a proof-of-concept study to evaluate the effectiveness of our test in inducing cough reflex response in HC and ABI patients, further RCT studies are needed for assessing the reliability of our method with respect to others [25]. Moreover, a direct comparison between our cough test (spray stimulation on the tongue) and another well-validated approach (nebulizer) is mandatory. However, it should be considered that the rationale behind the choice of using this kind of cough stimulation test is twofold: (a) to prevent irritation of the nasal and oral mucous membranes using a natural compound, and (b) to apply the selected amount of capsaicin with greater accuracy.

## 5. Conclusions

In this study, we provide preliminary evidence on the effectiveness of a new kind of simple and timesaving cough reflex induction method using a capsaicin commercial spray stimulation. This finding might lead to the development of a novel form of therapy exploiting the reflexive cough modulation. Indeed, spray capsaicin could be used in clinical practice not only to diagnose cough reflex alterations but also for rehabilitation purposes. The next stage would be to compare the cough response caused by capsaicin to that of other well-known toxigenic drugs, like citric acid, and validate the results in other independent groups of ABI patients.

## Figures and Tables

**Table 1:** Demographic and clinical characteristics of ABI patients at admission.

	ABI without Dysphagia (*n* = 26)	ABI with Dysphagia (*n* = 24)	*p*-Value
Age (years)	59.1 ± 18.3	51.6 ± 17.4	*t* = 1.46; n.s.
Gender (% Male)	17 (65%)	15 (63%)	X^2^ = 0.8; n.s.
Length of Stay in ICU (days)	24.2 ± 15.3	37.1 ± 17.7	*t* = 2.4 *p* < 0.02
Side of Lesion			
Left %	19 (73%)	7 (29%)	X^2^ = 10.1; *p* < 0.006
Right %	3 (12%)	10 (42%)	
Bilateral %	4 (15%)	7 (29%)	
Etiology			
Vascular %	22 (84%)	13 (54%)	X^2^ = 5.1; *p* = 0.06
Traumatic %	2 (8%)	8 (33%)	
Others %	2 (8%)	3 (13%)	
Route of Feeding (%)			
Oral feeding	24 (92%)	8 (33%)	X^2^ = 6.4; *p* < 0.04
nasogastric tubes	2 (8%)	5 (21%)	
PEG	0 (0%)	11 (46%)	
% Tracheostomy (yes)	1 (4%)	23 (96%)	X^2^ = 98.1; *p* < 0.001
% Infratentorial Lesion (yes)	5 (19%)	5 (21%)	n.s.
Barthel Index at admission	13.2 ± 20.1	2.7 ± 9.4	*t* = 2.89; *p* < 0.006
Barthel Index at discharge	56.3 ± 33.3	28.3 ± 35.1	*t* = 2.34; *p* < 0.02
GUSS scale	17.7 ± 2.5	8.9 ± 4.2	*t* = 8.98; *p* < 0.001

PEG: percutaneous endoscopic gastrostomy; ICU: Intensive Care Unit.

**Table 2:** Cough reflex response in HCs and ABI patients.

	Healthy Controls (*n* = 150)	ABI without Dysphagia (*n* = 26)	ABI with Dysphagia (*n* = 24)	*p*-Level
% response at 1° Administration	104 (69%)	20 (77%)	12 (50%)	X^2^ = 8.3; *p*= 0.21
% response at 2° Administration	14 (9%)	4 (15%)	5 (21%)	
% response at 3° Administration	8 (5%)	0 (0%)	4 (16%)	
Absence of response	24 (16%)	2 (8%)	3 (13%)	
Cough Reflex Trigger time (s)	12.2 ± 13.2	12.1 ± 7.5	23.8 ± 20.1	F = 3.42; *p* = 0.049
Cough Intensity	1.3 ± 0.5 1 [0–3]	1.3 ± 0.6 1 [0–3]	1.5 ± 0.6 1 [0–3]	n.s.

n.s.: Not significant.

## Data Availability

The data that support the findings of this study are available on request from the corresponding authors.
